# Implementation Status and Usability of Digital Health Interventions Among Health Care Workers and End Users at the Primary Health Care Level in Chandigarh, North India: Cross-Sectional Study

**DOI:** 10.2196/69824

**Published:** 2025-08-25

**Authors:** Munita Jat, Madhu Gupta, Shankar Prinja

**Affiliations:** 1 Department of Community Medicine and School of Public Health, Post Graduate Institute of Medical Education and Research, RN Dogra Block, Sector 12, Chandigarh, 160012, India, 91 7009769629

**Keywords:** digital health interventions, DHIs, health care workers, implementation, usability

## Abstract

**Background:**

Digital health interventions (DHIs) refer to the use of information and communication technologies to support or facilitate the achievement of health objectives. The Government of India has launched various DHIs at the primary health care level to improve health services and health-seeking behaviors. However, there is a paucity of evidence on the effectiveness of implementing these interventions and the user response from target end-users within the government health system setting.

**Objective:**

This study aimed to assess the implementation status of DHIs and the user response of target end users, that is, the general population and health care workers (HCWs), in health and wellness centers (HWCs) in Chandigarh, India.

**Methods:**

A cross-sectional study was conducted to assess the implementation status of 9 DHIs: the Electronic Vaccine Intelligence Network (eVIN), Reproductive and Child Health (RCH), Health Management Information System (HMIS), HWC portal, Comprehensive Primary Health Care–Noncommunicable Disease (CPHC-NCD), Family Planning–Logistics Management Information System (FP-LMIS), eSanjeevani, Integrated Disease Surveillance Program–Integrated Health Information Program (IDSP-IHIP) portal, Aarogya Setu, and the COVID-19 Vaccine Intelligence Network (CoWIN) app. Data were collected from 4 purposively selected HWCs using a pretested data extraction form and observation checklist from June to September 2022. The implementation status of these DHIs was evaluated by categorizing indicators into input, process, and output components and estimating cumulative percentage scores using a score-based logic model framework. Pretested interview schedules were used to assess awareness and user response of DHIs among 120 target end users (clients visiting HWCs) and 120 HCWs (auxiliary nurse midwives, data entry operators, and medical officers). The prevalence of user response was then estimated.

**Results:**

The implementation status scores of the eVIN and RCH portals ranged from 70% to 90%. The HMIS portal, HWC portal, CPHC-NCD portal, and FP-LMIS scored between 25% and 50%, while eSanjeevani and the IDSP-IHIP portal scored between 51% and 70%. Community awareness of DHIs was poor, ranging from 1% to 18.3%, except for Aarogya Setu (94/120, 78.3%) and the CoWIN app (43/120, 35.8%), despite 86.7% (104/120) of participants having access to a mobile phone. Low awareness of DHIs was significantly associated with lower socioeconomic status (*P*=.02) and lower education levels (*P*=.04). In total, 66% (80/120) of HCWs reported that working with DHIs was easy; however, 89.2% (107/120) stated that dual data entry increased their workload. Frequent technical glitches were most commonly reported for the Auxiliary Nurse Midwife OnLine app (78/80, 97%) by HCWs. Help desk or feedback options in DHIs were rarely used by auxiliary nurse midwives/multipurpose workers (0%‐3.8%).

**Conclusions:**

The RCH and eVIN portals were effectively implemented, eSanjeevani was moderately implemented, while the HMIS, HWC portal, CPHC, and FP-LMIS were poorly implemented. Community awareness of DHIs was low, except for the Aarogya Setu and CoWIN apps. Although HCWs found DHIs easy to use, increased workload due to dual data entry and frequent technical issues was a key concern.

## Introduction

Primary health care is essential health care made universally accessible to individuals and families in the community by means acceptable to them, through their full participation, and at a cost that the community and country can afford, as per the World Health Organization (WHO) [1]. One of the 4 principles of primary health care is the use of appropriate technology, defined by WHO as “methods, procedures, technologies, and equipment which are scientifically validated, adapted to local needs, acceptable to users as well as recipients, and maintainable with local resources” [1]. The term digital health was coined as a “broad comprehensive term including eHealth, that is, the application of information and communication technology in health and health-related field, mobile health (the use of mobile and wireless technologies for medical care), as well as emerging areas such as the application of advanced computing sciences in “big data and artificial intelligence” [2]. Digital health interventions (DHIs) are services delivered electronically through formal or informal care, such as electronic medical health records, health management information systems (HMISs), mobile apps, and telemedicine. [3] In low- and middle-income countries, including India, the imbalance between demand and supply of health care services can be addressed through DHIs, as they can act as catalysts to provide affordable, accessible, and equitable health care services. Recognizing this, the Government of India embarked on the journey of digital health, as evident in the National Health Policy (NHP), National eHealth Authority (NeHA), and the National Digital Health Blueprint (NDHB). A flagship program, the National Digital Health Mission (NDHM), was launched on August 15, 2020, with a vision of establishing a digital ecosystem to achieve universal health coverage [4,5]. Over the past decade, the Ministry of Health and Family Welfare (MoHFW) has taken various digital initiatives for health information management, tracking of beneficiaries, establishing online inventory, and supply chain management. These also include platforms or mobile apps made to seek patient feedback*,* improve health literacy, and enhance the use of health services [6,7]. Recently, due to COVID-19, there was a major boost in the adoption of telemedicine services such as eSanjeevani OPD [8]. The Aarogya Setu mobile app, launched during the pandemic, also achieved the remarkable milestone of reaching 50 million downloads in just 13 days, making it the fastest-growing app globally [9]. As reported in various studies, the use of DHIs in primary health care has shown promising results in areas such as mental health issues, tobacco cessation programs, maintaining the vaccine supply chain, and hypertension management [10-12]. However, the implementation status of these DHIs at the primary health care level remains uncertain. A systematic review of previous studies reported barriers such as infrastructure and technical limitations, psychological and personal factors, and concerns regarding increasing working hours, which challenge the successful adoption of DHIs [13,14]. DHIs, as a tool to achieve universal health coverage, can be leveraged only if there is greater demand and use. This depends on factors such as the level of awareness among the community [15], clients’ or health care providers’ interaction with DHIs, and their belief that using technology will positively affect health [16]. However, there is little evidence on end-user perception (eg, satisfaction and motivation) of DHIs at the primary health care level, or awareness among the population in Chandigarh. The majority of available studies have evaluated either an independent intervention for a single condition or were part of nongovernmental organization or isolated projects. There is a paucity of evidence in the existing literature regarding the status of government-launched DHIs’ implementation at the primary care level and the usability of these interventions among the community and health care workers (HCWs) in Chandigarh, India. Hence, this study was planned with the objectives to assess the implementation status of existing government-launched DHIs in public primary health centers and to ascertain the usability, in terms of user response, of DHIs by target end users (general population and clients) and HCWs while delivering health service in primary health centers in Chandigarh. The results of this study might guide program managers regarding the status and scope for improvement of the digitization of health interventions at the primary care level.

## Methods

### Operational Definitions

Operational definitions as per the WHO guidelines for monitoring and evaluation of DHIs are given below [16]:

1. Digital health: “The use of digital, mobile, and wireless technologies to support the achievement of health objectives. Digital health describes the general use of information and communication technologies for health and is inclusive of both mHealth and eHealth.”
2. Usability: “The degree to which a product or system can be used by specified users to achieve specified goals with effectiveness, efficiency, and satisfaction in a specified context of use.” In this study, only part of usability was evaluated in terms of user response.
3. Users or target end users: “The individuals who directly employ the technology using their mobile phones or wireless devices either to deliver health services or to receive services.”

### Study Design and Settings

A cross-sectional study was conducted in Chandigarh, a union territory with an estimated population of 1,364,000 (2024). Approximately 97% of the population resides in urban areas. Chandigarh has 2 tertiary health care centers, 1 district hospital, 1 subdivisional hospital, 2 urban community health centers, and 34 health and wellness centers (HWCs; including 5 Ayurveda, Yoga and Naturopathy, Unani, Siddha, and Homeopathy centers) [17]. To assess the implementation status of existing government-launched DHIs, public primary health centers were selected. To ascertain the usability of DHIs in terms of user response by target end users, face-to-face interviews were conducted, and questions were administered in person to members of the general population visiting the study HWCs, as well as to the HCWs delivering health services in primary health centers in Chandigarh.

### Study Health Facilities

To assess the implementation of DHIs, study health facilities were HWCs. Under the Ayushman Bharat Scheme, existing civil dispensaries and subcenters were upgraded to HWCs (recently renamed as Ayushman Arogya Mandir) [18]. These were established to provide universally accessible, free-of-cost, comprehensive primary health care. The primary focus is on promoting well-being and offering an extended range of services within the community. The comprehensive range of services now includes care in pregnancy and childbirth, neonatal and infant health care, adolescent health care, family planning, reproductive health care, management of communicable and noncommunicable diseases through National Health Programs, general outpatient care for acute simple illnesses, basic oral health care, care for common ophthalmic and ENT problems, elderly and palliative health care, emergency medical services, and screening with basic management of mental health ailments [17,18].

### Study DHIs

Through a preliminary exercise, we enlisted health apps or portals that were implemented at HCWs and used by HCWs. For the general population, we enlisted health apps and portals that were launched for them and were functional. DHIs (apps or portals) that were included in the study were (1) Comprehensive Primary Health Care–Noncommunicable Disease (CPHC-NCD) portal; (2) Reproductive, and Child Health (RCH) portal; (3) Auxiliary Nurse Midwife OnLine (ANMOL) app; (4) Electronic Vaccine Intelligence Network (eVIN); (5) Family Planning–Logistics Management Information System (FP-LMIS); (6) eSanjeevani OPD; (7) Integrated Disease Surveillance Program–Integrated Health Information Program (IDSP-IHIP) portal; (8) Ayushman Bharat–Health and Wellness portal (AB-HWC portal); (9) HMIS portal; (10) Ni-kshay portal or app, (11) COVID-19 Vaccine Intelligence Network (CoWIN); (12) Arogya Setu app; (13) National Health Portal; and (14) My Health Records. The list of DHIs to assess the implementation and HCWs’ and clients’ responses is given in Table S1 in Multimedia Appendix 1.

### Study Population

To assess the response of target end users, clients aged 18 years or older, irrespective of gender, who visited the study HWCs either seeking care for illness or as attendants, were contacted. HCWs, including data entry operators (DEOs), medical officers, auxiliary nurse midwives (ANMs), multipurpose workers (MPWs), and pharmacists, who were directly involved in the use and maintenance of DHIs, were enrolled in the study.

### Sample Size

The sample size for assessing usability among both target end users and HCWs was calculated using the formula: n=4pq/e^2^, where p=prevalence of usability of DHIs, which was assumed to be 50% (since there was a paucity of sufficient evidence in the existing literature on the prevalence of usability given the heterogeneity of selected DHIs, it was assumed to be 50% to estimate the sample size), q=(1p), and e=margin of error, assumed to be 10%. Considering a nonresponse rate of 20%, the final sample size was calculated as 120 for each group (target-end users and HCWs).

### Sampling Technique

The criteria for the selection of HWCs were the mean number of patients seen per day. A list of HWCs with a mean number of patients seen per day was made, and 2 HWCs with high, 1 with moderate, and 1 with low patient footfall were randomly selected (a total of 4 HWCs). For target end-user enrollment, the catchment area of 1 HWC with moderate footfall was selected. Beneficiaries or persons who were visiting the facility and who consented were interviewed in person consecutively until the sample size was achieved. Similarly, HCWs in HWCs who were available and consented were interviewed in person consecutively. For this, we visited randomly selected 11 HWCs of Chandigarh.

### Data Collection Tools and Methods

The data collection tools included a pretested observation checklist and data extraction form (Tool 1) to assess the implementation status of the DHIs, and pretested interview schedules (paper-based) for target end users (Tool 2) and HCWs (Tool 3) to assess the usability of DHIs. Tool 1 dealt with information on the functionality of DHIs, including technical factors such as connectivity, power, maintenance, data entry frequency, use, and management of currently operational DHIs. Portal-specific real-time reporting status, number of stock-out events, and number of clients enrolled under DHIs in the health facilities from April to June 2022 were collected. Data quality was checked by comparing the information recorded in the register (hard copies) with the corresponding data entries on the portal (DHI). The interview schedule for clients (Tool 2) was pretested in a health center in Haryana. During pretesting, it was found that there was low awareness of DHIs in the population except for the Aarogya Setu app, so questions were modified to first assess the awareness regarding DHIs. Tool 2 dealt with collecting information on the sociodemographic status of clients, language literacy, source of seeking health information, awareness of DHIs (ever heard of or aware of the mentioned DHIs), and whether they have visited portals or downloaded any mobile app from the listed DHIs. Data were collected using paper-based forms and entered into Epicollect 5 [19]. Tool 3 was used to collect information about the background characteristics of HCWs, the digital ecosystem in the working area, language literacy, training on DHIs, and reported responses on the usability of DHI with options such as “easy to use,” “motivated/intending to use DHIs,” “satisfaction with DHIs,” and “several minutes/hours spent daily on the portal.” Responses from Tool 3 were entered in an internet-based format (Google Forms).

### Data Analysis

Data were entered in Microsoft Excel, and analysis was done using SPSS (trial version 25.0, IBM Corp) [20]. Descriptive statistics such as numbers and frequencies were calculated for awareness, apps downloaded, language proficiency, and user response in terms of ease of use, user satisfaction, and motivation. The chi-square test was applied to assess the association between sociodemographic factors and awareness of DHIs among clients. A *P*<.05 was considered significant. A logical framework model having input, process, and output indicators was used to assess the implementation status of the DHIs. Care was taken while deciding on these indicators from the review of literature, respective DHI guidelines, and public health expert opinions. A score-based system was developed to categorize the implementation status of DHIs. The scoring criteria for each indicator using the logic model are given in Table S2 in Multimedia Appendix 1. After scoring each indicator, cumulative scores of DHIs were calculated in all 3 categories—that is, input, process, and output indicators—and measured in percentages. Equal weight was given to each category. The status of DHI implementation, as per percentage cumulative scores obtained (ie, cumulative scores obtained divided by maximum cumulative scores×100), was categorized as follows: “not implemented=0%‐24%,” “poorly implemented=25%‐50%,” “fairly implemented=51%‐70%,” “effectively implemented=71%‐90%,” and “very effectively implemented=100%‐91%.”

### Ethical Considerations

For this observational cross-sectional study, ethical clearance was obtained from the Institute’s Ethics Committee (IEC), Postgraduate Institute of Medical Education and Research (PGIMER), on March 17, 2022 (reference no IEC-INT/2022/MPH-59). Permission was obtained from the Chandigarh administration for the participation of HCWs. All ethical criteria were fulfilled before, during, and after data collection. Informed consent was obtained from participants before the interviews using a written informed consent form. A participant information sheet was given to all the participants before participation in the study. Personal information of participants was kept confidential, and there was no breach of confidentiality. The study data were anonymous. As this was an observational study, no compensation was provided, as per IEC guidelines. No identification of individual participants or users in any images in the paper or Multimedia Appendix was possible.

## Results

### Implementation Status of DHIs at the Primary Care Level

Regarding the implementation status of DHIs, eVIN and RCH were found to be effectively implemented (70%‐90%), whereas ANMOL was not implemented. HMIS, HWC, CPHC-NCD app, and FP-LMIS were found to be poorly implemented (25%‐50%). eSanjeevani and IDSP-IHIP portal fall in the category of fairly implemented (51%‐70%). HWC and HMIS were not operational in 2 HWCs because DEOs were not positioned at these centers. FP-LMIS and CPHC-NCD were in the poorly implemented category, as they were not operational due to technical issues or lack of training. Although inputs were available for the ANMOL app, it was not operational in any HWC because of technical issues. Regarding maturity level, all DHIs were in the mature category except the FP-LMIS app, eSanjeevani, and CPHC-NCD app, which were at the scale-up level (Table 1).

**Table 1.** Overall implementation and maturity status of 9 DHIs^a^ at 4 health and wellness centers, obtained through data extraction sheet and observation checklist at the primary care level in Chandigarh, 2022.

Name of DHI	Total scores obtained per HWCs^b^				Sum of total score obtained/sum of maximum score	Cumulative score, %	Maturity stage	Inference
	HWC 1	HWC 2	HWC 3	HWC 4				
RCH^c^ portal	24	21	24	24	93/116	80	6	Matured and effectively implemented
ANMOL**^d^**	3	3	3	3	12/64	18	6	Matured but not implemented
eVIN^e^	23	21	24	22	90/104	86	6	Matured and effectively implemented
IDSP-IHIP portal^f^	14	13	14	19	60/86	69	6	Matured and fairly implemented
FP-LMIS^g^ app	10	1	6	2	19/52	36	5	Scale-up and poorly implemented
HMIS portal^h^	20	21	0	0	41/96	42	6	Matured and poorly implemented
HWC portal	19	18	0	0	37/96	38	6	Matured and poorly implemented
eSanjeevani	7	7	8	9	31/52	59	5	Scale-up and fairly implemented
CPHC-NCD app^i^	6	3	10	14	33/84	39	5	Scale-up and poorly implemented

a DHI: digital health intervention.

b HWC: health and wellness center.

c RCH: Reproductive and Child Health.

d ANMOL: Auxiliary Nurse Midwife Online.

e eVIN: Electronic Vaccine Intelligence Network.

f IDSP-IHIP: Integrated Disease Surveillance Program–Integrated Health Information Platform.

g FP-LMIS: Family Planning–Logistics Management Information System.

h HMIS: Health Management Information System.

i CPHC-NCD: Comprehensive Primary Health Care–Noncommunicable Disease.

### Awareness and Users’ Response Among Target End Users

Out of 120 target end users, 33.3% (40/120) were in the age group of 18‐30 years and 30‐45 years, and 86.7% (104/120) of participants owned a smartphone. Around 90% (108/115) of those with access reported having an adequate internet connection. There was low awareness (ever heard of DHIs or knew something about the respective DHI) of DHIs (range: 1%-18.3%; 1/120 to 22/120), except for Aarogya Setu (94/120, 78.3%) and the CoWIN app (43/120, 35.8%) in the community. Out of 94 participants, 56 (46.7%) had downloaded the Aarogya Setu app (Table 2). Clients with low socioeconomic status and low education were significantly more likely to be unaware of DHIs (*P*=.02 and *P*=.04, respectively). There was no statistically significant association between age, sex, and awareness of DHIs (Table 3). Since the awareness of DHIs was very low, we limited our questions to awareness only, and user response was not explored.

**Table 2.** Background characteristics of clients (target end users) visiting health and wellness centers, Chandigarh, 2022.

Parameter	Frequency (N=120), n (%)
Age (years)	
18‐30	40 (33.3)
31‐45	40 (33.3)
46‐60	15 (12.5)
>60	25 (20.8)
Sex	
Male	71 (59.16)
Female	49 (40.83)
Socioeconomic status	
Upper	11 (9.2)
Upper middle	57 (47.5)
Lower middle	32 (26.7)
Upper lower	20 (16.7)
Education	
Illiterate or up to primary school	10 (8.3)
Up to high school or a diploma	55 (45.5)
Graduate or above	55 (45.5)
Area of residence	
Urban	78 (65.0)
Rural	3 (2.5)
Slum or slum rehabilitation	39 (32.5)
Occupation	
Unemployed or elementary work	75 (62.0)
Craft, trade-related work, or clerks	24 (19.8)
Professionals or managers	21 (17.4)
Experiencing from health condition (multiple responses, n=131)	
Hypertension	27 (22.5)
Tuberculosis	3 (2.5)
DM^a^	19 (15.8)
None of the above	79 (65.8)
Others	3 (2.5)
Language easily understood for health information (multiple responses, n=258)	
English	81 (67.5)
Hindi	120 (100.0)
Punjabi	57 (47.5)
Sources to receive health information (multiple responses, n=235)	
Mass media (including social media, Newspapers, and Television)	122 (51.9)
Public health facility	92 (39.1)
Web browsing	19 (8.1)
Others	2 (0.9)
Have smartphones that they use	104 (86.7)
Adequate internet connection (n=115)	108 (90.0)
Awareness of DHIs^b^	
CoWIN^c^	43 (35.8)
Aarogya Setu	94 (78.3)
eSanjeevani OPD	22 (18.3)
National Health Portal	12 (10.0)
My Health Records	1 (0.8)
None of the above	19 (15.8)
Downloaded app	
CoWIN	6 (5)
Aarogya Setu	56 (46.7)
eSanjeevani OPD	3 (2.5)
Visited portal	
NHP^d^ portal	3 (2.5)

a DM: diabetes mellitus.

b DHI: digital health intervention.

c CoWIN: COVID-19 Vaccine Intelligence Network.

d NHP: National Health Portal.

**Table 3.** Awareness of DHIs^a^ among clients (target end users) by age, sex, education, and socioeconomic status at health and wellness centers, Chandigarh, 2022.

Parameter	Awareness of DHIs (aware of any DHI), n (%)			*P* value
	Yes (n=101)	No (n=19)	Total (N=120)	
Age group (years)				.85
18‐30	33 (82.5)	7 (17.5)	40 (100)	
31‐45	35 (87.5)	5 (12.5)	40 (100)	
46‐60	13 (86.7)	2(13.3)	15 (100)	
>60	20 (80.0)	5 (20.0)	25 (100)	
Sex				.25
Male	62 (87.3)	9 (12.7)	71 (100)	
Female	39 (79.6)	10 (20.4)	49 (100)	
Area of residence				.07
Urban	70 (89.7)	8 (10.3)	78 (100.0)	
Rural	2 (66.7)	1 (33.3)	3 (100.0)	
Slum or slum rehabilitation	29 (74.4)	10 (25.6)	39 (100.0)	
Education				.04
Illiterate or up to primary school	6 (60.0)	4 (40.0)	10 (100.0)	
Up to middle school or diploma	45 (81.8)	10 (18.2)	55 (100.0)	
Graduate or above	50 (90.9)	5 (9.1)	55 (100.0)	
Socioeconomic status				.02
Upper	8 (72.7)	3 (27.3)	11(100.0)	
Upper middle	54 (94.7)	3 (5.3)	57 (100.0)	
Lower middle	25 (78.1)	7 (21.9)	32 (100.0)	
Upper lower	14 (70.0)	6 (30.0)	20 (100.0)	

a DHI: digital health intervention.

### Status of HCWs’ Usability

Nearly 49.2% (59/120) of HCWs were aged 31 to 40 years, 87.5% (105/120) were females, 66.6% (80/120) were working as ANMs and MPWs, 5.8% (7/120) as DEOs, and 10% (12/120) as medical officers (Table 4).

**Table 4.** Background characteristics of health care workers and reported or perceived digital ecosystem at primary health care level, Chandigarh, 2022.

Parameter	Number (N=120), n (%)
Age group (years)	
21‐30	27 (22.5)
31‐40	59 (49.2)
41‐50	21 (17.5)
51‐60	13 (10.8)
Sex	
Male	15 (12.5)
Female	105 (87.5)
Education level	
High school	14 (11.7)
Graduate or diploma	75 (62.5)
Postgraduation	31 (25.8)
Designation	
ANM^a^	67 (55.8)
MPW^b^	13 (10.8)
Staff nurse or LHV^c^	1 (0.8)
DEO^d^	7 (5.8)
Medical officer	12 (10)
STS^e^ or NTEP^f^	8 (6.7)
Others	12 (10)
Language literacy (multiple responses, n=291)	
English	120 (100)
Hindi	107 (89.2)
Punjabi	64 (53.3)
DHIs^g^ used by HCWs^h^ (multiple responses, n=503)	
RCH^i^ portal	82 (68.3)
ANMOL^j^ portal	80 (66.7)
Ni-kshay	9 (7.5)
IDSP IHIP^k^ portal	87 (72.5)
eVIN^l^	48 (40.0)
FP-LMIS^m^	42 (35.0)
HWC^n^ portal	11 (9.2)
CoWIN^o^	7 (5.8)
CPHC-NCD^p^	71 (59.2)
HMIS^q^	11 (9.2)
eSanjeevani	15 (12.5)
Other (PMMVY^r^ and eHospital)	40 (33.3)
Perceived adequacy: adequate number of computers/tablets at the health facility center	
Yes	102 (85.0)
Strength or speed of internet at health facility	
No connectivity	4 (3.3)
Very poor	22 (18.3)
Poor	38 (31.7)
Satisfactory	49 (40.8)
Strong	7 (5.8)
Very strong	0 (0)
Ease of working on digital device as compared with paper system	
Very difficult	0 (0)
Difficult	23 (19.2)
Equal	17 (14.2)
Easy	74 (61.7)
Very easy	6 (5)
Agree that dual data entry has increased your workload	
Strongly agree	94 (78.3)
Agree	13 (10.8)
Neutral	2 (1.7)
Disagree	3 (2.5)
Strongly disagree	1 (0.8)
Preferred language for web portals or mobile health apps	
English	115 (95.8)
Hindi	5 (4.2)
Punjabi	0 (0)

a ANM: auxiliary nurse midwife.

b MPW: multipurpose worker.

c LHV: lady health visitor.

d DEO: data entry operator.

e STS: Senior Treatment Supervisor.

f NTEP: National Tuberculosis Elimination Program.

g DHI: digital health intervention.

h HCW: health care worker.

i RCH: Reproductive and Child Health.

j ANMOL: Auxiliary Nurse Midwife OnLine.

k IDSP IHIP: Integrated Disease Surveillance Platform–Integrated Health Information Platform.

l eVIN: Electronic Vaccine Intelligence Network.

m FP-LMIS: Family Planning–Logistics Management Information System.

n HWC: health and wellness center.

o CoWIN: COVID-19 Vaccine Intelligence Network.

p CPHC-NCD: Comprehensive Primary Health Care–Noncommunicable Disease.

q HMIS: Health Management Information System.

r PMMVY: Pradhan Mantri Matru Vandana Yojana.

In terms of the digital ecosystem, 85% (102/120) of participants reported an adequate number of computers or tablets at the workplace (perceived adequacy), and 41%(49/120) reported having satisfactory internet strength. In total, 66% (80/120) of HCWs rated working on DHIs as “easy,” and 89.2% (107/120) agreed that dual data entry has increased their workload. English (115/120, 95%) was the most preferred language while working on DHIs (Table 5).

**Table 5.** Usability of digital health interventions by health care workers at health and wellness centers (HWCs), Chandigarh, Union Territory, 2022.

Parameter	RCH^a^ portal	ANMOL^b^	Ni-kshay	eVIN^c^	IDSP-IHIP^d^	CPHC-NCD^e^ app	eSanjeevani	FP-LMIS^f^	HMIS^g^	HWC portal
Using it on daily or weekly basis, n (%)	85 (100)	80 (100)	9 (100)	82 (100)	101 (100)	94 (100)	15 (100)	82 (100)	—^h^	12 (100)
Yes, n (%)	82 (96.5)	17 (21.3)	9 (100)	47 (57.3)	82 (81.2)	70 (74.5)	15 (100)	40 (48.7)	10 (100)	8 (66.7)
Easy to use, n	82	78	9	47	82	70	15	40	10	8
Very difficult, n (%)	0 (0)	59 (75.6)	0 (0)	0 (0)	1 (1.2)	2 (2.9)	0 (0)	0 (0)	0 (0)	0 (0)
Difficult, n (%)	6 (7.3)	11 (14.1)	1 (11.1)	1 (2.12)	1 (1.2)	10 (14.3)	3 (20)	0 (0)	2 (20)	0 (0)
Equal, n (%)	7 (8.5)	4 (5.1)	1 (11.1)	1 (2.12)	6 (7.3)	22 (31.4)	3 (20)	0 (0)	3 (30)	0 (0)
Easy, n (%)	52 (63.4)	4 (5.1)	3 (33.3)	5 (10.6)	31 (37.8)	28 (40)	7 (46.7)	6 (15)	3 (30)	2 (25)
Very easy, n (%)	17 (20.7)	0 (0)	4 (44.4)	41 (87.2)	43 (52.4)	8 (11.4)	2 (13.3)	34 (85)	2 (20)	8 (75)
Easy to navigate different pages or task, n	82	78	9	47	82	70	15	40	10	8
Strongly disagree, n (%)	3 (3.7)	35 (53)	0 (0)	0 (0)	1 (1.2)	0 (0)	1 (6.7)	0 (0)	0 (0)	0 (0)
Disagree, n (%)	9 (11.0)	11 (16.7)	1 (11.1)	0 (0)	4 (4.9)	11 (15.7)	4 (26.7)	0 (0)	2 (20)	0 (0)
Neutral, n (%)	6 (7.3)	8 (12.1)	3 (33.3)	0 (0)	6 (7.3)	29 (41.4)	3 (20)	1 (2.5)	1 (10)	0 (0)
Agree, n (%)	56 (68.3)	10 (15.2)	3 (33.3)	12 (25.5)	38 (46.3)	23 (32.9)	4 (26.7)	11 (27.5)	6 (60)	4 (50)
Strongly agree, n (%)	8 (9.8)	2 (3)	2 (22.2)	35 (74.5)	33 (40.2)	7 (10)	3 (20)	28 (70)	1 (10)	4 (50)
Total minutes spent on data entry/working on portal in a day, n	82	17	9	47	82	70	15	40	10	8
0‐30 minutes, n (%)	0 (0)	0 (0)	3 (33.3)	47 (100)	82 (100)	5 (7.1)	0 (0)	40 (100)	7 (70)	8 (100)
30‐60 minutes, n (%)	25 (30.5)	7 (41.2)	4 (44.4)	0 (0)	0 (0)	25 (35.7)	8 (53.3)	0 (0)	0 (0)	0 (0)
60‐90 minutes, n (%)	19 (23.2)	4 (23.5)	2 (22.2)	0 (0)	0 (0)	8 (11.4)	3 (20)	0 (0)	0 (0)	0 (0)
>90 minutes, n (%)	38 (46.3)	6 (35.3)	0 (0)	0 (0)	0 (0)	32 (45.7)	4 (26.7)	0 (0)	3 (30)	0 (0)
Received training, n^i^	82	78	9	47	82	70	15	40	10	8
Yes, n (%)	66 (80.5)	78 (97.5)	8 (88.9)	45 (80.5)	79 (78.2)	70 (74.5)	8 (53.3)	37 (74.5)	7 (70)	5 (62.5)
No of days received training, n	66	78	8	45	79	70	8	37	6	5
1‐2 days, n (%)	64 (97)	75 (96.2)	6 (75)	45 (100)	77 (97)	70 (100)	8 (100)	37 (100)	6 (85.7)	5 (100)
3‐4 days, n (%)	2 (3)	3 (3.8)	2	0 (0)	2 (3)	0 (0)	0 (0)	0 (0)	1 (14.3)	0 (0)
1 week, n (%)	0 (0)	0 (0)	2 (25)	0 (0)	0 (0)	0 (0)	0 (0)	0 (0)	0 (0)	0 (0)
Used help desk or feedback option, n	82	78	9	47	82	70	15	40	10	8
Yes, n (%)	0 (0)	3 (3.8)	4 (44.4)	3 (6.4)	2 (2.4)	1 (1.4)	0 (0)	0 (0)	0 (0)	0 (0)
No, n (%)	82 (100)	76 (96.2)	5 (55.5)	44 (93.6)	80 (97.6)	69 (98.6)	15 (100)	40 (100)	10 (100)	8 (100)
Technical glitches while using portal, n	82	78	9	47	82	70	15	40	10	8
Never, n (%)	0 (0)	0 (0)	0 (0)	34 (70.8)	20 (24.4)	5 (7.1)	0 (0)	29 (72.5)	0 (0)	2 (25)
Rarely, n (%)	15 (18.3)	0 (0)	2 (22.2)	14 (29.2)	27 (32.9)	21 (30)	3 (20)	10 (25)	2 (20)	0 (0)
Sometimes, n (%)	47 (57.3)	2 (2.5)	4 (44.4)	0 (0)	18 (22)	23 (32.9)	7 (46.7)	1 (2.5)	5 (50)	2 (25)
Most of the time, n (%)	18 (22.0)	4 (5.0)	3 (33.3)	0 (0)	13 (15.9)	21 (30)	5 (33.3)	0 (0)	3 (30)	3 (37.5)
Always, n (%)	2 (2.4)	74 (92.5)	0 (0)	0 (0)	4 (4.9)	0 (0)	0 (0)	0	0 (0)	1 (12.5)
Clearly understand the language of portal, n	82	78	9	47	82	70	15	40	10	8
Yes, n (%)	81 (98.8)	76 (97.4)	8 (88.9)	46 (97.9)	80 (97.6)	70 (100)	15 (100)	40 (100)	5 (50)	8 (100)
Few words difficult to understand, n (%)	1 (1.2)	0 (0)	1 (11.1)	1 (2.1)	2 (2.4)	0 (0)	0 (0)	0 (0)	5 (50)	0 (0)
Perceive portal as useful										
Yes, n (%)	75 (91.5)	5 (6.3)	8 (88.9)	44 (93.6)	29 (35.4)	25 (35.7)	12 (80)	35 (87.5)	5 (50)	8 (100)
Not sure, n (%)	5 (6.1)	0 (0)	1 (11.1)	2 (4.3)	38 (46.3)	37 (52.9)	1 (6.7)	4 (10)	2 (20)	0 (0)
No, n (%)	2 (2.4)	75 (93.8)	0 (0)	1 (2.1)	15 (18.3)	8 (11.4)	2 (13.3)	1 (2.5)	3 (30)	0 (0)
Satisfied with functioning of portal, n	82	78	9	7	82	70	15	40	10	8
Yes, n (%)	56 (68.3)	1 (1.3)	5 (55.6)	44 (93.6)	56 (68.3)	25 (35.7)	5 (33.3)	37 (92.5)	5 (50)	5 (62.5)
Not sure, n (%)	17 (20.7)	61 (78.2)	2 (22.2)	3 (6.4)	14 (17.1)	28 (40)	7 (46.7)	3 (7.5)	4 (40)	2 (25)
No, n (%)	9 (11.0)	16 (20.5)	2 (22.2)	0 (0)	12 (14.6)	17 (24.3)	3 (20)	0 (0)	1 (10)	1 (12.5)
Motivated to use portal										
Yes, n (%)	77 (93.9)	1 (1.3)	9 (100)	44 (93.6)	26 (31.7)	15 (21.4)	6 (40)	32 (80)	6 (60)	8 (100)
Not sure, n (%)	2 (2.4)	1 (1.3)	0 (0)	3 (6.4)	26 (31.7)	34 (48.6)	5 (33.3)	8 (20)	3 (30)	0 (0)
No, n (%)	3 (3.7)	78 (97.5)	0 (0)	0 (0)	30 (36.6)	21 (30)	4 (26.7)	0 (0)	1(10)	0 (0)
Has helped in adhering to treatment protocol, n	82	78	9	47	82	70	15	40	10	8
Yes, n (%)	58 (70.7)	5 (6.3)	9 (100)	41 (87.2)	20 (24.4)	30 (42.9)	11 (73.3)	32 (80)	5 (50)	3 (37.5)
Sometimes, n (%)	19 (23.2)	11 (13.8)	0 (0)	2 (4.3)	46 (56.1)	22 (31.4)	3 (20)	5 (12.5)	5 (50)	5 (62.5)
No, n (%)	5 (6.1)	64 (80.0)	0 (0)	4 (8.5)	16 (19.5)	18 (25.7)	1 (6.7)	3 (7.5)	0 (0)	0 (0)

a RCH: Reproductive and Child Health.

b ANMOL: Auxiliary Nurse Midwife Online.

c eVIN: Electronic Vaccine Intelligence Network.

d IDSP-IHIP: Integrated Health Information Platform.

e CPHC-NCD: Comprehensive Primary Health Care–Noncommunicable Disease.

f FP-LMIS: Family Planning–Logistics Management Information System.

g HMIS: Health Management Information System.

h Not available.

i Where n is the total number of participants who received training for the app or portal among the total users.

ANMs and MPWs reported disagreement in most of the parameters for the ANMOL portal (78/80, 97%), mainly due to technical glitches. It was reported that even after entering data on the app, the data were not synced with the RCH portal. Therefore, they had to maintain similar details of one beneficiary at three places, that is, the RCH register, the RCH portal, and the ANMOL app. Triple data entry was the reason for lack of satisfaction (1/80, 1.3%), low motivation (1/80, 1.3%), and low perceived usefulness (5/80, 6.3%) of the ANMOL portal among ANMs. In total, 46.3% (38/85) of HCWs reported spending >90 minutes per day on entering data on the RCH portal. eSanjeevani, currently in the early phase of implementation, was used by medical officers (n=15). In addition, 60% (9/15) of them rated it as “easy to use,” but 33.3% (5/15) reported having technical issues (slow internet speed) most of the time. Help desk or feedback options were rarely used by health care providers for any of the DHIs. The most often cited reason for limited use of the feedback or help desk option was delayed responses. It was informed that verbal communication with superiors was the most common way to raise queries or provide feedback on DHIs. Technical issues were reported with all of the DHIs except for eVIN (34/82, 70% never; 14/82, 30% rarely). HCWs clearly understood the language of DHIs except for HMIS (5/10, 50%). ANMs or MPWs reported the IDPS portal (S form) as “easy to use” (74/80, 92%), and 66% (53/80) were satisfied with the functioning of the portal, albeit only 27% (23/80) of ANMs were motivated to use it. The primary reason was the belief among ANMs or MPWs that their primary duty was to provide MCH services, and that activities related to disease surveillance should be delegated to other staff (Table 5).

## Discussion

### Principal Findings

The results of this study highlighted that the RCH portal and eVIN app were effectively implemented; the IDSP-IHIP portal and eSanjeevani were fairly implemented, the HMIS portal, HWC portal, CPHC-NCD portal or app, and FP-LMIS were weakly implemented; and the ANMOL app was not operational in the study HWCs. There was very low awareness among clients (general population and beneficiaries) regarding DHIs launched by the Government of India to improve health literacy and health-seeking behavior (except for Aarogya Setu and the CoWIN app). This low awareness was found to be significantly associated with low socioeconomic status and low education level. The majority of the HCWs agreed on having an adequate digital ecosystem (number of computers, tablets, and connectivity) and considered operating data online to be easy. However, they reported increased workload due to dual data entry. Despite having a similar ecosystem at health care facilities, health care providers’ responses to each DHI varied depending upon technical issues, the time consumed to enter data, and individual factors.

Effective implementation of eVIN at the primary health care level is similar to a recent study that reported improvement in vaccines cold chain management after implementation of eVIN [21]. This could be because of the better user interface of eVIN or the smaller number of data elements to be entered on the eVIN portal. The RCH portal, although effectively implemented, needed improvement in its output indicators. Although the RCH portal was supposed to be a cloud-based system, data interoperability was up to the state level only, while its usage was at the primary care level. A beneficiary in the second or third trimester migrating to a UT needed to reregister herself again on the portal. This was one of the reasons for the inability to achieve full registration within the first trimester. A study conducted in Uttar Pradesh and Rajasthan on the assessment of an earlier version of the RCH portal, that is, the Mother and Child Tracking System (MCTS) portal, reported poor data quality assessment (overall performance numbers were 34% for pregnant women and 33% for children in Rajasthan, while performance numbers were 18% for pregnant women and 25% for children in Uttar Pradesh) [22]. However, a study on the MCTS portal in the Ambala district of Haryana reported that HCWs perceived that MCTS has ensured better data quality [23]. Although HMIS has evolved into a robust system for health information management up to the primary level, due to a lack of dedicated DEOs, reports were sent in paper-based format to the district health administration at 2 HWCs. A study on the implementation status of HMIS in hospitals of central Ethiopia reported overall implementation as poor with a 56% overall percentage score [24]. It was even lower, that is, 42% in our study. The parameters used in the Ethiopia study (2021) were availability, compliance, and use of HMIS in tertiary care hospitals.

At the community level, this study reported low awareness of DHIs among clients despite having a smartphone and adequate internet connection, except for the Aarogya Setu app and CoWIN. This could be because of the enhanced awareness during the COVID-19 pandemic in the community due to widespread mass marketing of the Aarogya Setu app and regulations mandating its download while traveling during the pandemic [25]. Around 46.7% (56/120) of participants had downloaded the Aarogya Setu app in this study. Responses of users on usefulness (53%), easy to use (70%), and satisfaction with functionality (50%) were similar to a review study by Kodali et al [26], which reported higher satisfaction level (>50%), acceptance rate (80%), and perceived usefulness (72.8%) as important parameters among clients for DHI usability. A study conducted in Singapore on awareness of the COVID-19 pandemic and adoption of digital health services in urban Asian populations reported a high level of awareness of COVID-19 infection but low acceptance of digital health services. They also reported that older and lower-income participants were less likely to use digital health services [27]. This finding is similar to our study regarding lower awareness among people from low socioeconomic status; however, no significant association was noted with age. These results were also consistent with a systematic literature review by Reiners et al [28], which explored sociodemographic factors affecting the use of eHealth. It was reported that people with lower income, less education, and those living in rural areas were less likely to use eHealth. Tailored delivery of eHealth was recommended to improve usage [28]. Hence, to increase awareness and empower Indian citizens with DHIs, a push in terms of awareness camps and counseling at health facilities is needed.

Use of feedback or help desk option for the HCWs was limited despite facing several technical issues in running the portals, such as the ANMOL app and the RCH portal. This might be due to a defective or underperforming feedback resolution mechanism, leading to HCWs rarely using it in the future. Despite the majority of participants perceiving data entry into the digital system as easy, HCWs reported (89%, 107/120) an increase in workload due to dual data entry (both in manual and digital form) and considered working on DHIs to be extremely time-consuming. A study on the usage of a health information portal at the primary health care level in Haryana also reported an increased workload of health workers as a result of additional time spent on entering physical records. There were limited opportunities for using the data produced at the primary health care level in web-based platforms [29]. Another study conducted in Uttar Pradesh and Rajasthan on the assessment of the MCTS portal [22], and in Ambala district, Haryana [23], reported overburdened HCWs due to data documentation, which was identified as a barrier to implementation of MCTS.

### Strengths

This is the first study to assess the implementation status of DHIs at the primary health care level using a logic framework approach and WHO guidelines on monitoring and evaluation of DHIs in Chandigarh, India. Using input, process, and output parameters helped in identifying implementation gaps at these levels and providing specific recommendations that will strengthen the implementation of the DHIs at the primary care level. This study has also looked into the awareness and usability of digital health initiatives from the clients’ perspective to improve health literacy, awareness, and health service usage. The sample size and sampling technique for assessing the usability of the HCWs are representative of Chandigarh city, and hence those results can be generalizable to the city.

### Limitations

There might be limitations in deciding the input, process, and output indicators, which might influence the implementation status of the DHIs. However, care was taken while deciding on these indicators from the review of literature, respective DHIs’ guidelines, and public health experts’ opinions. Due to time constraints and feasibility issues, the implementation status of DHIs was assessed in 4 centers; hence, selection bias cannot be ruled out, which may limit the external validity. Each of these DHIs needs further exploration on implementation, barriers, and facilitators specific to a program by conducting a qualitative study and including a greater number of HWCs for the generalizability of results. The generalizability of the results pertaining to the clients’ usability and awareness regarding DHIs is limited to the study area only. Due to feasibility and time constraints, we aimed only for descriptive analysis. The usability of DHIs by health care providers and associated factors can be further assessed more objectively in future studies.

### Conclusions

Effective implementation and use are important for the sustainability and positive impact of DHIs. It is important to assess and monitor the currently operational DHIs in terms of inputs, processes, and outputs to identify gaps in implementation and ways to improve them. Also, how routinely HCWs use DHIs and their response to use will eventually affect the output, outcome, and impact of DHIs. As the Government of India is moving forward to a digitized health care system, it is important to know the awareness of DHIs at the community level, as they are the end-target users and receivers of digital health services. This study assessed the perspective of target-end users of DHIs. Awareness, acceptance, and demand for DHIs among marginalized people are of utmost importance for reducing health inequality and improving their health status. That will eventually help to achieve the Sustainable Development Goals and universal health coverage. The results of this study might guide policy makers to identify gaps and improve the implementation of DHIs by conducting enhanced training and timely refresher training sessions on the use or incentivization of HCWs, developing targeted awareness campaigns, or technical support initiatives. Further, adopting technological innovations such as automating data entry or improving system interfaces, along with a robust feedback or issues-resolution system, can minimize technological barriers. We recommend that there is a need for further in-depth assessment of barriers to implementation, awareness, and adoption of poorly performing government-launched DHIs, as well as evaluating long-term outcomes such as sustained adoption rates and improvement in health care delivery. This study also suggests the need to develop behavior-change communication interventions to increase the demand and uptake of DHIs in the community.

## Supplementary material

10.2196/69824 Multimedia Appendix 1 List of digital health interventions (DHIs) included in the study to assess implementation, health care worker, and client responses, and the indicators with corresponding scores used to assess the implementation status of DHIs in primary health care facilities.

## Abbreviations

ANM: auxiliary nurse midwife
ANMOL: Auxiliary Nurse Midwife OnLine
CoWIN: COVID-19 Vaccine Intelligence Network
CPHC-NCD: Comprehensive Primary Health Care–Noncommunicable Disease
DEO: data entry operator
DHI: digital health intervention
eVIN: Electronic Vaccine Intelligence Network
FP-LMIS: Family Planning–Logistics Management Information System
HCW: health care worker
HMIS: Health Management Information System
HWC: health and wellness center
IDSP-IHIP: Integrated Disease Surveillance Program–Integrated Health Information Platform
MCTS: Mother and Child Tracking System
MPW: multipurpose worker
NDHB: National Digital Health Blueprint
NDHM: National Digital Health Mission
NeHA: National eHealth Authority
NHP: National Health Policy
RCH: Reproductive and Child Health
WHO: World Health Organization
