# Supplementary material for: Implementation Status and Usability of Digital Health Interventions Among Health Care Workers and End Users at the Primary Health Care Level in Chandigarh, North India: Cross-Sectional Study
Source: JMIR Form Res. 2025 Aug 25;9:e69824. doi: 10.2196/69824 (PMC12377784; doi:10.2196/69824)
Supplement: Multimedia Appendix 1 [file formative-v9-e69824-s001.docx]

Table S1. The list of DHIs included in the study assessed the implementation, HCWs, and clients’ response.

| To assess the implementation status | To assess the HCWs’ response | To assess the client's response |
| --- | --- | --- |
| 1. CPHC_NCD App 2. RCH portal 3. ANMOL 4. eVin 5. FP_LIMS 6. eSanjeevani 7. IDSP_IHIP Portal 8. AB-HWC App 9. HMIS portal | 1. RCH portal 2. ANMOL 3. eVIN 4. FP_LIMS 5. Ni-kshay portal 6. IDSP_IHIP portal 7. CPHC_NCD app for ANM 8. HMIS 9. CoWIN 10. AB-HWC app 11. eSanjeevani | 1. CoWin app 2. Arogya Setu app 3. eSanjeevani OPD 4. National Health Portal 5. My health records |

Table S2: List of indicators and the corresponding scores to assess the implementation status of DHIs in primary health care facilities.

| Name of DHIs | Status of input indicators (score range) | Status of process indicators  (score range) | Status of output indicators  (score range) |
| --- | --- | --- | --- |
| 1. RCH portal | - Number of HCWs who enter data on the portal (0-1). - % of HCWs trained in data entry (0-1) - Status of portal updation (0-1) - Validation checks in place for missing essential data entry (0-1) | - Number of days in a week when data entry is done (0-1) - Number of supervisory visits conducted (0-2) - % Forms filled in RCH register (1-5) - % Data accuracy on comparing the RCH register to RCH portal (1-5) - % Of HCWs received refresher training (0-1) | - % Pregnant women registered on RCH portal during reference period (1-5) - % ANC registration who were within the 1st trimester during the reference period (1-5) - Number of HCWs received work plan (0-1) |
| Total score range: 0-29 | 0-4 | 0-14 | 0-11 |
| 2. ANMOL portal | - Number of HCWs who enters data (0-1) - % Of HCWs who received training (0-1) - Number of tablets/ANM (0-1) - Validation checks in place for missing essential data entry (0-1) | - Number of days for data entry is done in a week (0-1) - Status of frequency of supervisory visits (0-2) - % Data accuracy on comparing ANMOL to RCH register (0-5) | - % Of beneficiaries registered using ANMOL app (1-5) - Number of HCWs generated work plan using ANMOL (0-1) |
| Total score range: 0-16 | 0-4 | 0-7 | 0-5 |
| 3. IHIP_IDSP portal | - Number of HCWs who enters data for P form (0-1)   and s form) (0-1)   - % of HCWs who received training (0-1) - Status of portal updation (0-1) - Validation check in place for missing essential data entry (0-1) | - Number of days for data entry is done in a week (0-1) - % of days with data entry error during reference period (0-5) - Status of frequency of supervisory visits (0-2) - % of HCWs received refresher training for IHIP (0-1) | - Number of outbreak events from April to June 2022 (IDSP portal) (0-1) - % of P forms send during reference period (1-5) - % of S forms send during the reference period (1-5) |
| Total score range: 0-25 | 0-5 | 0-9 | 0-11 |
| 4. eVIN | - Number of HCWs who enters data (0-1) - % of HCWs who received training (0-1) - Number of functional temperature loggers (0-1) - Status of portal updation (0-1) - Alert for nearly stock out/ expiry of vaccines (0-1) | - Number of days when data is entered in a week (0-1) - % of data entry error (1-5) - % sessions for which indents were filled completely in register (1-5) - Status of supervisory visit (0-2) - Whether refresher training provided (0-1) | - Number of stock-out events of vaccine in reference period (0-1) - Number of events of vaccine wastage due to poor cold chain management (0-1) - % of events when stocks were updated timely on portal during reference period (0-5) |
| Total score range: 0-26 | 0-5 | 0-14 | 0-7 |
| 5. FP-LIMS  Portal | - Number of HCWs who enters data on portal (0-1) - % of HCWs who received training (0-1) - Status of portal updation (0-1) - Alert for nearly stock out/ expiry of FP logistics (0-1) | - 0% data entry error from register to portal (0-5) - Whether Supervisory visits are conducted (0-2) - Whether refresher training provided (0-1) | - 0% events of stock-out of FP logistics (0-1) - % of events when stocks were updated timely on portal during reference period {was not measured due to data unavailability} |
| Total score range: 0-13) | 0-4 | 0-8 | 0-1 |
| 6.HWC portal | - Number of HCWs who enters data on portal (0-1) - % of HCWs who received training (0-1) - Status of portal updation (0-1) - Validation check in place for missing essential data entry(0-1) | - Number of days in a week for data entry (0-1) - % forms filled completely in monthly report (0-5) - % of HCP received refresher training (0-1) - Whether Supervisory visits are conducted (0-2) | - Data is same on Cross-verification of common data elements with HMIS report (0-1) - % of daily report generated during reference period (1-5) - % of monthly report generated during reference period (1-5) |
| Total score range: 0-24 | 0-4 | 0-9 | 0-11 |
| 7. HMIS portal | - Number of HCWs who enters data on portal (0-1) - % of HCWs who received training (0-1) - Status of portal updation (0-1) - Validation check in place for missing essential data entry (0-1) | - Number of days in a week for data entry (0-1) - % forms filled in monthly report (1-5) - Status of Supervisory visits (0-2) - % of HCP received refresher training (0-1) | - Data element is same on Cross-verification of common data elements with HWC report (0-1) - % of daily report generated during reference period (1-5) - % of monthly report generated during reference period (1-5) |
| Total score range: 0-24 | 0-4 | 0-9 | 0-11 |
| 8. eSanjeevaniOPD | - Number of HCWs who enters data on portal (0-1) - % of HCWs who received training (0-1) - Status of portal updation (0-1) - Validation check in place for missing essential data entry (0-1) | - Number of days in a week for data entry (0-1) - Availability of monthly/weekly report or offline record keeping (0-1) - Whether OTP received during each consultation (0-1) - Status of Supervisory visits (0-2) | - Number of teleconsultations during reference period (1-4) |
| Total score range: 0-13 | 0-4 | 0-5 | 0-4 |
| 9. CPHC_NCD portal | - Number of HCWs who enters data on portal 1) ANM portal (0-1)   2)MO portal (0-1)   - % of HCWs who received training (0-1) - Status of portal updation (0-1) - Validation check in place for missing essential data entry (0-1) | - Number of days in a week for data entry in ANM portal (0-1) - Number of days in a week for data entry in MO portal (0-1) - % of HCP received refresher training (0-1) - Status of supervisory visits (0-2) | - % eligible population enrolled on portal (1-5) - % of population who were examined at facility during reference period (1-5) - Whether HCWs use the due list/area to be covered in the portal (0-1) |
| Total score range: 0-21 | 0-5 | 0-5 | 0-11 |
